# Supplementary material for: Prevalence and Risk Factors of Infection in the Representative COVID-19 Cohort Munich
Source: Int J Environ Res Public Health. 2021 Mar 30;18(7):3572. doi: 10.3390/ijerph18073572 (PMC8038115; doi:10.3390/ijerph18073572)
Supplement: Supplementary file 1 [file ijerph-18-03572-s001.pdf]

Supplementary Materials for

Prevalence and Risk Factors of Infection in the Representative COVID-19 Cohort Munich

Michael Pritsch [1,2,†]; Katja Radon [3,4,5,†]; Abhishek Bakuli [1,†]; Ronan Le Gleut [6,7,†]; Laura Olbrich [1,2]; Jessica Michelle Guggenbüehl Noller [1]; Elmar Saathoff [1,2]; Noemi Castelletti [1]; Mercè Garí [6]; Peter Pütz [6,8]; Yannik Schälte [6,9]; Turid Frahnow [6,8]; Roman Wölfel [2,10]; Camilla Rothe [1]; Michel Pletschette [1]; Dafni Metaxa [1]; Felix Forster [3,5]; Verena Thiel [1]; Friedrich Rieß [1,2]; Maximilian Nikolaus Diefenbach [1]; Günter Fröschl [1,4]; Jan Bruger [1]; Simon Winter [1]; Jonathan Frese [1]; Kerstin Puchinger [1]; Isabel Brand [1]; Inge Kroidl [1,2]; Jan Hasenauer [6,9,11,†]; Christiane Fuchs [6,7,8,9,†]; Andreas Wieser [1,2,†]; Michael Hoelscher [1,2,4,*,†] and on behalf of the KoCo19 study group [‡]

1 Division of Infectious Diseases and Tropical Medicine, University Hospital, LMU Munich, 80802 Munich, Germany; pritsch@lrz.uni-muenchen.de (M.P.); bakuli@lrz.uni-muenchen.de (A.B.); olbrich@lrz.uni-muenchen.de (L.O.); guggenbuehl@lrz.uni-muenchen.de (J.M.G.N.); saathoff@lrz.uni-muenchen.de (E.S.); castelletti@lrz.uni-muenchen.de (N.C.); rothe@lrz.uni-muenchen.de (C.R.); Michel.Pletschette@lrz.uni-muenchen.de (M.P.); metaxa@lrz.uni-muenchen.de (D.M.); Verena.Thiel@lrz.uni-muenchen.de (V.T.); Friedrich.Riess@lrz.uni-muenchen.de (F.R.); diefenbach@lrz.uni-muenchen.de (M.N.D.); froeschl@lrz.uni-muenchen.de (G.F.); jan.bruger@med.uni-muenchen.de (J.B.); simon.winter@med.uni-muenchen.de (S.W.); jonathan.frese@med.uni-muenchen.de (J.F.); kerstin.puchinger@med.uni-muenchen.de (K.P.); isabel.brand@med.uni-muenchen.de (I.B.); ikroidl@lrz.uni-muenchen.de (I.K.); wieser@mvp.lmu.de (A.W.)

2 German Center for Infection Research (DZIF), Partner Site Munich, 80802 Munich, Germany; RomanWoelfel@Bundeswehr.org

3 Institute and Outpatient Clinic for Occupational, Social and Environmental Medicine, University Hospital, LMU Munich, 80336 Munich, Germany; katja.radon@med.uni-muenchen.de (K.R.); Felix.Forster@med.uni-muenchen.de (F.F.)

4 Center for International Health (CIH), University Hospital, LMU Munich, 80336 Munich, Germany

5 Comprehensive Pneumology Center (CPC) Munich, German Center for Lung Research (DZL), 89337 Munich, Germany

6 Institute of Computational Biology, Helmholtz Zentrum München—German Research Center for Environmental Health, 85764 Neuherberg, Germany; ronan.legleut@helmholtz-muenchen.de (R.L.G.); merce.gari@helmholtz-muenchen.de (M.G.); peter.puetz@uni-bielefeld.de (P.P.); yannik.schaelte@helmholtz-muenchen.de (Y.S.); turid.frahnow@helmholtz-muenchen.de (T.F.); jan.hasenauer@uni-bonn.de (J.H.); christiane.fuchs@helmholtz-muenchen.de (C.F.)

7 Core Facility Statistical Consulting, Helmholtz Zentrum München—German Research Center for Environmental Health, 85764 Neuherberg, Germany

8 Faculty of Business Administration and Economics, Bielefeld University, 33615 Bielefeld, Germany

9 Center for Mathematics, Technische Universität München, 85748 Garching, Germany

10 Bundeswehr Institute of Microbiology, 80937 Munich, Germany

11 Interdisciplinary Research Unit Mathematics and Life Sciences, University of Bonn, 53113 Bonn, Germany

* Correspondence: hoelscher@lrz.uni-muenchen.de; Tel: +49-89-44005-9801

† Those authors contributed equally.

‡ KoCo19 study group: Emad Alamoudi, Jared Anderson, Valeria Baldassare, Maximilian Baumann, Marc Becker, Marieke Behlen, Jessica Beyerl, Rebecca Böhnlein, Anna Brauer, Vera Britz, Friedrich Caroli, Lorenzo Contento, Alina Czwienzek, Flora Deák, Emma Dech, Laura Dech, Jana Diekmannshemke, Anna Do, Gerhard Dobler, Jürgen Durner, Ute Eberle, Judith Eckstein, Tabea M. Eser, Philine Falk, Volker Fingerle, Stefanie Fischer, Marius Gasser, Sonja Gauder, Otto Geisenberger, Christof Geldmacher, Leonard Gilberg, Kristina Gillig, Philipp Girl, Elias Golschan, Vitus Grauvogl, Celina Halfmann, Tim Haselwarter, Arlett Heiber, Matthias Herrmann, Stefan Hillmann, Christian Hinske, Janna Hoefflin, Tim Hofberger, Michael Höfinger, Larissa Hofmann, Sacha Horn, Kristina Huber, Christian Janke, Ursula Kappl, Charlotte Kiani, Isabel Klugherz, Norah Kreider, Arne Kroidl, Magdalena Lang, Clemens Lang, Silvan Lange, Ekaterina Lapteva, Michael Laxy, Reiner Leidl, Felix Lindner, Alexander Maczka, Alisa Markgraf, Paula Matcau, Rebecca Mayrhofer, Anna-Maria Mekota, Hannah Müller, Katharina Müller, Leonie Pattard, Claire Pleimelding, Stephan Prückner, Konstantin Pusl, Elba Raimúndez, Julius Raschka, Jakob Reich, Raquel Rubio-Acero, Nicole Schäfer, Paul Schandelmaier, Lara Schneider, Sophie Schultz, Mirjam Schunk, Lars Schwettmann, Heidi Seibold, Paul Stapor, Jeni Tang, Fabian Theis, Sophie Thiesbrummel, Eva Thumser, Niklas Thur, Julian Ullrich, Julia Waibel, Claudia Wallrauch, Julia Wolff, Pia Wullinger, Tobias Würfel, Patrick Wustrow, Houda Yaqine, Sabine Zange, Eleftheria Zeggini, Thorbjörn Zimmer, Thomas Zimmermann, Lea Zuche.

Contents

Text S1.	Sampling design and corrections of seroprevalence.	p 3
Text S2.	Details on cut-offs and validation.	p 5
Text S3.	Handling of missing data and risk factor analysis.	p 6
Text S4.	Influence of household size and household clustering.	p 7
Figure S1.	Validation of classifier based on Ro-N-Ig results.	p 8
Figure S2.	Comparison of primary test results (Ro-N-Ig vs EI-S1-IgG) with other tests.	p 9
Figure S3.	Number of participants per city district (A), unweighted (upper figure) and weighted (lower figure) prevalence (%) of Ro-N-Ig seropositive samples per city district (B) with lower (C) and upper (D) 95% Confidence Intervals (CI).	p 10
Figure S4.	SARS-CoV-2 seroprevalence obtained by different classifiers for different specificities and sensitivities.	p 11
Figure S5.	Missing patterns observed in our complete analysis data.	p 12
Figure S6.	Missing patterns observed in our multiple regression analysis data with important covariates only.	p 13
Figure S7.	Roche N pan-Ig seroprevalence in the KoCo19 study population unweighted (top) and weighted (bottom).	p 14
Figure S8.	Sampling probabilities for Munich constituencies.	p 15
Figure S9.	Fraction of individuals with positive Ro-N-Ig result for different household sizes.	p 16
Table S1.	Individual characteristics of the KoCo19-study participants comparing telephone interviewees to those answering the online questionnaire.	p 17
Table S2.	Prevalence of Roche N pan-Ig seropositivity and risk factor analysis adjusted for age and sex using three different methods by individual, household and health characteristics of the KoCo19 study population.	p 18
Table S3.	Multivariate risk factor analysis for SARS-CoV-2 seropositivity mutually adjusted for all variables in the table.	p 21
Table S4.	Classifier's specificity and sensitivity depending on the manufacturer's or the optimised cut-offs.	p 22
Table S5.	Overview on the household size distribution, the number of tested individuals per household and the number of positive tests.	p 23

Online Text S1

Sampling design and corrections of seroprevalence. To obtain reliable and representative estimates of SARS-CoV-2 seroprevalence in the Munich population, we corrected the observed (crude) seroprevalence in our data, i.e. the number of positive cases divided by the sample size, for two characteristics: the sampling design underlying our cohort, and the sensitivity as well as specificity of tests used.

The population-based prospective cohort study KoCo19 is located in the city of Munich, Germany. From 5 April to 12 June 2020, we carried out the initial fieldwork in randomly selected households, using a two-stage sampling design. The initial study population of KoCo19 consists of the Munich general population 14 years and older.

The first stage of the sampling procedure refers to the selection of the 100 out of 755 constituencies. This selection was done via a rejective sampling design [1], initially with equal probabilities for each constituency to be included in the sample (~13%). The sample of 100 constituencies was checked to be a representative sample of the Munich population regarding the age structure, the percentages of the population with migration background, of households with children and households with only one member. A sample was considered representative if the respective mean fractions in the sample differed from the mean fractions across all 755 Munich constituencies by less than 10 percent points. Only samples of 100 constituencies that fulfilled these requirements had a non-zero probability to be selected. A Monte Carlo simulation using 5000 iterations for random samples of 100 constituencies showed inclusion probabilities at the constituency level ranging from 12%–15% (Figure S8), indicating that the rejection step does not induce considerable bias. Moreover, the regression estimator (which is equivalent to the calibrated estimator, see below) for the rejective sample (with inclusion probabilities between 12% and 15%) has similar properties to the regression estimator for the original selection procedure (simple random sampling with inclusion probabilities ~13%) [2], which facilitates the calculation of the variance and the associated confidence interval.

The second stage of the sampling procedure consists in the selection of approximately 30 households for each of the 100 drawn constituencies, totalling around 3000 households in the sample. These households were obtained via random routes starting in each selected constituency (which could be assimilated to a systematic sampling with equal probabilities[3] or to a simple random sampling). The random routes often crossed the borders of the constituencies, which means that a household could be included in the sample via its own constituency or via a neighbouring one. To account for these multiple ways to be included in the sample, we considered the first and second order neighbours (neighbours of a neighbour) for each selected constituency and applied a generalized weight share method[4] for the weights of the households.

Finally, all members 14 years and older were asked to donate blood samples. If participants refused to give blood samples, the sampling weights of the consenting participants within the same household were increased to represent the other members. In case the age of one or more members in the household was missing, we accounted for household structure (number of members, age of the members, etc.) to impute the missing age(s) by its mode (i.e. by the case with the most occurrences). This results in considering that in households with two members, the missing age is almost always one of another adult in the household, while in households with three or more members, the missing ages are usually concerning children in the household.

Once the sampling weights for all participants were computed, they were calibrated in order to fit to the Munich population regarding the sex and age structure, the percentages of the population with migration background, of households with children and single-person households (auxiliary information). Here, we employed the calibration technique by Deville et al [5]. The resulting calibrated estimator (e.g. the seroprevalence for the Munich population using calibrated weights) is asymptotically equivalent to the regression estimator.

The variance associated to this calibrated estimator can then be derived thanks to linearisation[6] and residual[5,6] techniques. In short, the variance of the calibrated estimator is asymptotically equivalent to the variance of the total of the residuals of a linear regression using the linearized variable as response and the auxiliary variables used in the calibration process as covariates. This variance estimation accounts for the different stages of the sampling design (inference on finite population), i.e. $V = V_1 + V_2$ with V_1 the variance associated to the selection of the constituencies and V_2 the one associated to the selection of the households. 95% confidence intervals could then be calculated. The normality of the Horvitz-Thompson estimator in case of a two-stage sampling design has been proven by Chauvet et al [7].

In Figure S4, we compare the estimated SARS-CoV-2 seroprevalence (and the associated 95% confidence interval) obtained according to the sampling design (using calibrated weights as described above) to the one without considering any weighting strategy. The variance associated to the weighted estimator was calculated as described above. For the variance of the unweighted estimator we used the classical formula $P(1-P)/n$ (inference on infinite population). To account for the fact that the target variable within households is correlated, the population size used for this variance was n = 2994, that is the number of households in the sample rather than n = 5313, the number of participants.

In addition to accounting for the sampling design, we also consider the probabilities of the laboratory tests to yield false negatives or false positive results. Following Sempos and Tian [8], we calculate the adjusted seroprevalence as $(\hat{p} + \widehat{sp} - 1)/(\widehat{sen} + \widehat{sp} - 1)$, where $\hat{p}$ is the crude seroprevalence (fraction of positive cases among the observed population), $\widehat{sp}$ the estimated specificity and $\widehat{sen}$ the estimated sensitivity. Whilst $(p + sp - 1)/(sen + sp - 1)$ with true probability p for a positive test result and true sensitivity and specificity sen and sp is an exact formula for the true seroprevalence, it is only approximate if $\hat{p}$, $\widehat{sp}$ are $\widehat{sen}$ are calculated and plugged in independently. For that reason, the case $\hat{p} + \widehat{sp} \leq 1$ can occur. In this case, the above formula would yield a negative adjusted seroprevalence estimate. For our data, this happened for EI-S1-IgG. Instead of reporting a negative adjusted seroprevalence, we set the value to zero although positive test results have been observed. This estimate has to be considered with care since the standard adjustment method is not fully applicable [9].

To summarize, we report SARS-CoV-2 seroprevalence in this manuscript calculated in four different ways:

- weighted: correcting for the sampling design for our study cohort using calibrated weights,
- unweighted: not correcting for the sampling design,
- adjusted: correcting for false positives and false negatives based on a test's sensitivity and specificity,
- unadjusted: not correcting for false test results.

Applying the weighted and adjusted Ro-N-Ig seroprevalence (1.82%; 95% CI: 1.28-2.37%) to the number of inhabitants aged 14 and older living in Munich (1,369,444) allows us to estimate the number of individuals who developed SARS-CoV-2 antibodies (24,990; 95% CI 17,584-32,396; using more than two digits for seroprevalence). The underreporting factor from Figure 3C varies depending on the share of the 6,293 officially registered PCR-positive cases in Munich who are living in private households. The calculation of the infection fatality ratio is similar, using the estimated number of infections and assuming a share of the 216 registered COVID-19 related deaths in Munich occurring in private households (see Figure 3F).

According to RKI, around 13% of the reported cases for COVID-19 in Germany occurred in institutions until the end of the study period [10]. We can thus consider that 87% of the infections occurred in private households. Assuming that this percentage is the same for Munich leads to an underreporting factor of 4.5. In this calculation we use the entire population size as an approximation for the number of people living in private households since only a small number lives in institutions and an adjustment does not change the rough estimate of 4.5. If all cases were registered in private households, this factor would be approximately equal to 4. The same calculation was done for the number of registered deaths: 46% of COVID-19 related deaths in Germany occurred in institutions as reported by RKI [10]. Assuming that 54% of the registered deaths in Munich occurred in private households leads to an IFR of 0.47%. If all deaths had occurred in private households, the IFR would be approximately equal to 0.86%.

In our survey, we asked participants about already known (and thus registered) SARS-CoV-2 infection status. Based on this information, we estimate 4367 (95% CI 1,952–6783) officially reported cases in private households in Munich. Using these numbers allows us to estimate the share of the 6,293 officially reported cases that occurred in private households (69%; 95% CI 31–100%) for Munich. Assuming that 69% of the registered cases occurred in private households leads to an underreporting factor of 5.7.

To facilitate reproducibility and reuse, the code used to perform the analyses and generate the figures was made available on GitHub (https://github.com/koco19/lab_epi) and has been uploaded to ZENODO (http://doi.org/10.5281/zenodo.4300922, accessed on 28 March 2021) for long-term storage.

Online Text S2

Details on cut-offs and validation. To assess the robustness of the seropositivity estimate obtained based on the Ro-N-Ig assay with an optimised cut-off, we compared it to seropositivity estimates obtained using other classifiers: Ro-N-Ig assay with the manufacturer's cut-off, EI-S1-IgG and EI-S1-IgA each with an optimised and the manufacturer's cut-off, and two machine learning techniques—namely a support vector machine and a random forest—using information from Ro-N-Ig, EI-S1-IgG and EI-S1-IgA. Detailed descriptions of the cut-off optimisation for the three assays, the training of the support vector machine and the random forest, as well as the estimation of the specificities and sensitivities of all classifiers are provided by Olbrich et al [11]. In short, an optimised cut-off for a single assay is the median of the cut-offs that classify best in 10000 bootstrap samples, each sampled with replacement from our set of 1266 individuals with known seropositivity status. In each bootstrap replication, the respective best cut-off is used to predict the serological status of the observations not included in the bootstrap sample (out-of-bag observations). After averaging the predictions for each observation over all bootstrap replications, the majority votes are chosen and compared to the true serological status to calculate specificity and sensitivity. For the support vector machine and the random forest, a similar approach is employed: After having optimised the tuning parameters, 2000 bootstraps and respective out-of-bag estimations are used to calculate specificity and sensitivity. The manufacturer's cut-off for a single assay is determined by the manufacturer based on its own data, and specificity and sensitivity are computed by us by comparing the true serological status to the predictions based on the cut-off for the entire sample of 1266 individuals. Bootstrapping this sample and predicting out-of-bag observations is also used to optimise the tuning parameters and estimate specificities and sensitivities of the support vector machine and the random forest.

Classification of test results according to optimized cut-offs are reported in Figures S1 and S2. Table S4 reports resulting test-sensitivity and -specificity for all classifiers. For the classifiers based on a single assay and the cut-off provided by the manufacturer, also one specificity and two sensitivities using manufacturer's information are given. In particular, the specificity chosen is the average specificity over all cohorts investigated by the manufacturer, while for the sensitivity scenarios of low sensitivity (assuming the time elapsed from a positive PCR test to the antibody test to be intermediate) and high sensitivity (using the highest category of elapsed time reported) are considered. This allows the assessment of the variability of the seroprevalence estimates when different measures of test performance are applied. As it is typical for low-prevalence settings, even small differences in specificity lead to fundamentally different results, as can be seen for both Euroimmun assays. Despite the variability in the results and independent of the application of weights, all classifiers indicate a low seropositivity with all point estimates and most of the upper bounds of the 95% bootstrap percentile confidence intervals being below 2.5% (Figure S4).

Online Text S3

Handling of missing data and risk factor analysis. The data collected in this study contained gaps. Overall, the data table was filled to 94.1%. Most missing values (5.9%) were due to non-response on the personal questionnaires. The serological data were complete for Ro-N-Ig, and there was only one missing observation for E1-S1-IgA/G. The missingness pattern and frequencies are illustrated in Figures S5 and S6. To handle the missing data, we performed multiple imputation as a way of sensitivity analysis and compare it to scenarios where imputation is not performed.

The standard method for analysis of binary outcomes for risk factor analysis is the framework of generalized linear models (GLM), in particular logistic regression analyses. However, in our study, the participants were clustered within the households as a sampled unit, and thus, we use the generalized linear mixed models (GLMM) [12,13].

We assume the missingness in our data to be missing at random (MAR), where the specification of a dropout model is not necessary. This allows us to make valid inference which is based on the likelihood function conditional on the observed data alone (complete case analyses). The GLMM is valid under the above conditions since the estimates are obtained by maximum likelihood. In the case of non-likelihood marginal models, the semi-parametric method of generalized estimating equations (GEE) is popularly used to model correlated non-Gaussian outcomes including missing outcomes. However, GEE would require the stronger condition of missingness completely at random (MCAR) and is not performed here [12,14].

We performed the imputation of the missing covariates to compare our estimates after imputation with the GLMM estimates. For this imputation of covariates under the assumption of the base model, we used the Joint Analysis and Imputation of Incomplete Data Framework (JointAI) in R to obtain the updated OR estimates and standard errors [12,14,15]. Under this framework, the estimation process is sequentially fully Bayesian, by modelling the GLMM of interest jointly with the incomplete covariates information. Joint analysis and imputation are performed simultaneously to allow for consistency in the parameter estimation process without the requirement for any pooling steps for the imputed data. One of the major advantages of the Bayesian approach is that the joint distribution of all data allows for the use of all available information of the outcome in the imputation of the incomplete covariates particularly in complex setups of clustered data analysis where covariates can be present in multiple levels of hierarchy.

For Table S2 we allowed for 100 burn-in/adaption samples followed by 500 MCMC iterations for each of the models considered. For Table S3 we allowed for 3000 burn-in/adaption samples and 12,000 MCMC iterations, respectively. Within each iteration, there is an imputed data set created and the regression coefficient(s) for the corresponding risk factor(s) of interest are estimated. The hyperparameters (non- informative) for the fixed effect regression coefficients are assumed to be Gaussian with mean 0 and standard deviation 0.01 whereas the variance components (1/sigma) are assumed to be Gamma distributed with shape and rate parameter being equal to 0.001.

Online Text S4

Influence of household size and household clustering. In total 93 participants of the KoCo19 study were tested positive. These individuals lived in households of different sizes, and there were 82 households in which at least one infection was observed. As household members below 14 years of age were excluded and as some household members did not agree to participate, only 76% of the members of all households were tested. While we had a large number of households with more than two household members, in most households only two persons were tested.

Interestingly, we observed that members of 1-person households were more likely to be tested positive than members of households of size 2, 3 and 4, while for large households the fraction of positive tests was again increasing (Table S2). This is in agreement with the OR of 1.54 computed by the risk factor analysis for 1-persons households, and might point towards different habits (Table S3). Yet, despite the higher infection risk in 1-person households, out of the 93 individuals with a positive test result, 22 lived in a household with another individual which was tested positive. Furthermore, out of the 66 households with more than one inhabitant in which at least one individual was tested positive, 33.33% had a second individual who was tested positive. Due to the low overall prevalence, both is unlikely in the absence of transmission within households. Accordingly, it indicates a substantial fraction of within-household transmissions.

To further study the household clustering, we performed a risk factor analysis using the GLMM framework (which used a logistic model). Using a likelihood ratio test (LRT), we investigated whether the additional variance component (random effect) due to the clustering within households in the GLMM compared to the GLM was significantly different from zero. We considered the null hypothesis $H_0 : D = 0$ versus the alternative $H_1 : D = d_{11}$ for some non-negative scalar d_{11}, where D is the variance of the random effect. The hypotheses were evaluated using the asymptotic null distribution for the difference between the likelihoods of the two models, $-2\,(\ln \lambda_{0\,RF} - \ln \lambda_{1\,RF}) \rightarrow \chi^2_{0:1}$ (mixture of χ^2_0 and χ^2_1 with equal weights 0.5) [12,16,17]. $\lambda_{0\,RF}$ is the likelihood under the GLM without any random effect, whereas $\lambda_{1\,RF}$ is the corresponding likelihood under the GLMM with one random effect for the intra-household clustering. For the analysis described in Table S3 under the frequentist framework we obtained the value for $-2\,(\ln \lambda_{0\,RF} - \ln \lambda_{1\,RF}) = 278.85$, and when compared to the mixture of χ^2_0 and χ^2_1, we obtain a p-value less than 0.001. This implied again that we have a significant clustering within households. In addition to this, in the Bayesian framework we estimated the random effect variance to be 7.13 with 95% Credible Interval from 3.4 to 12.4. All this evidence favoured the presence of positive clustering within households.

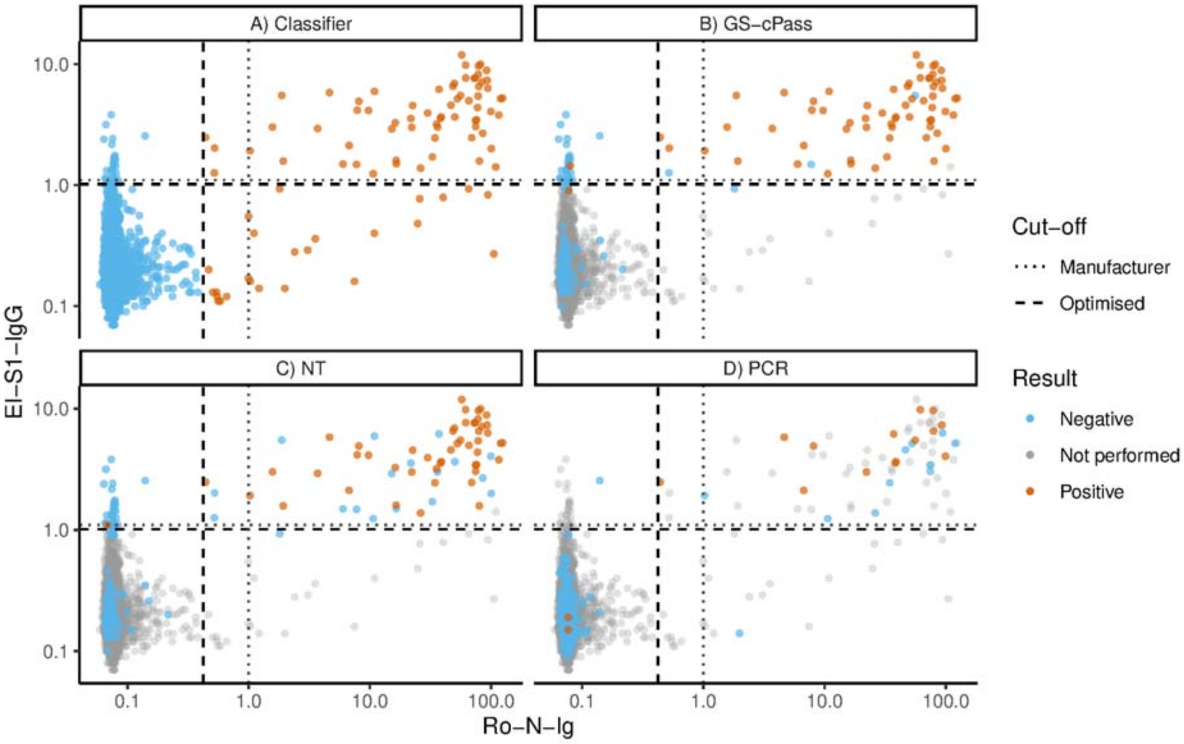

Online Figure S1. Validation of classifier based on Ro-N-Ig results. The subplots depict the results of the Ro-N-Ig and EI-S1-IgG results for 5313 participants. (**A**) Prediction of classifier based on Ro-N-Ig results using optimised threshold derived by Olbrich et al., (**B**) results using cPass test, (**C**) results of the neutralisation test, and (**D**) results of a PCR test [11].

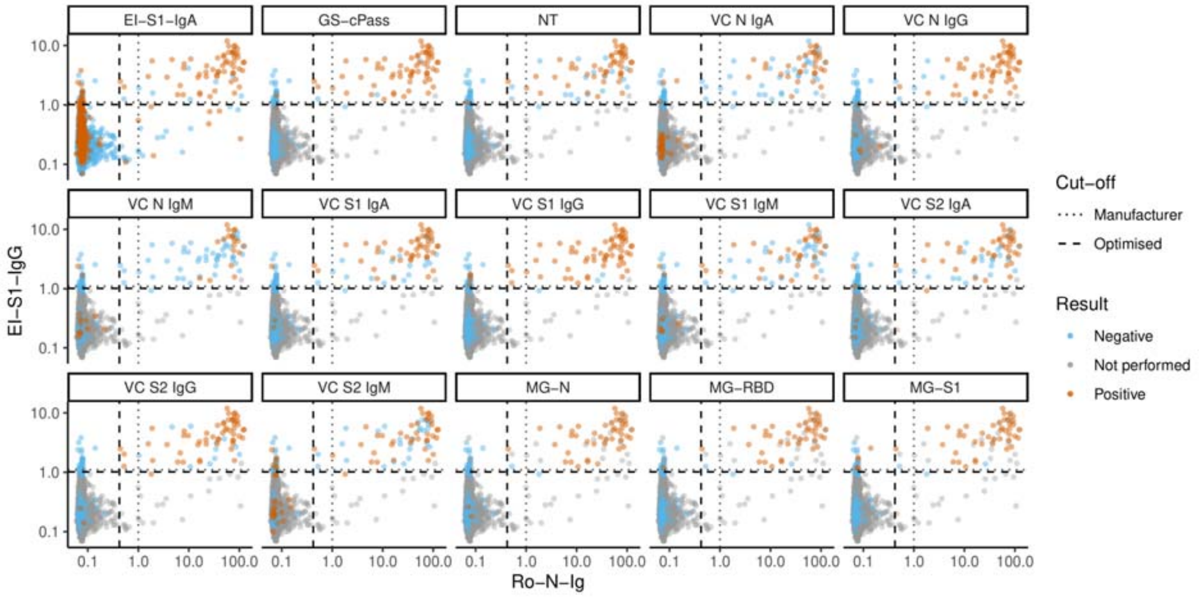

Online Figure S2. Comparison of primary test results (Ro-N-Ig vs EI-S1-IgG) with other tests. All plots show the same measurements of Ro-N-Ig values vs. EI-S1-IgG values on a continuous scale. Lines indicate whether they lie below or above the optimized (dashed) or the manufacturer's (dotted) cut-off for Ro-N-Ig (vertical) and EI-S1-IgG (horizontal). In each plot, colors represent the binary result of the test indicated in the respective title: blue for negative, orange for positive and grey for non-performed analysis of the respective test.

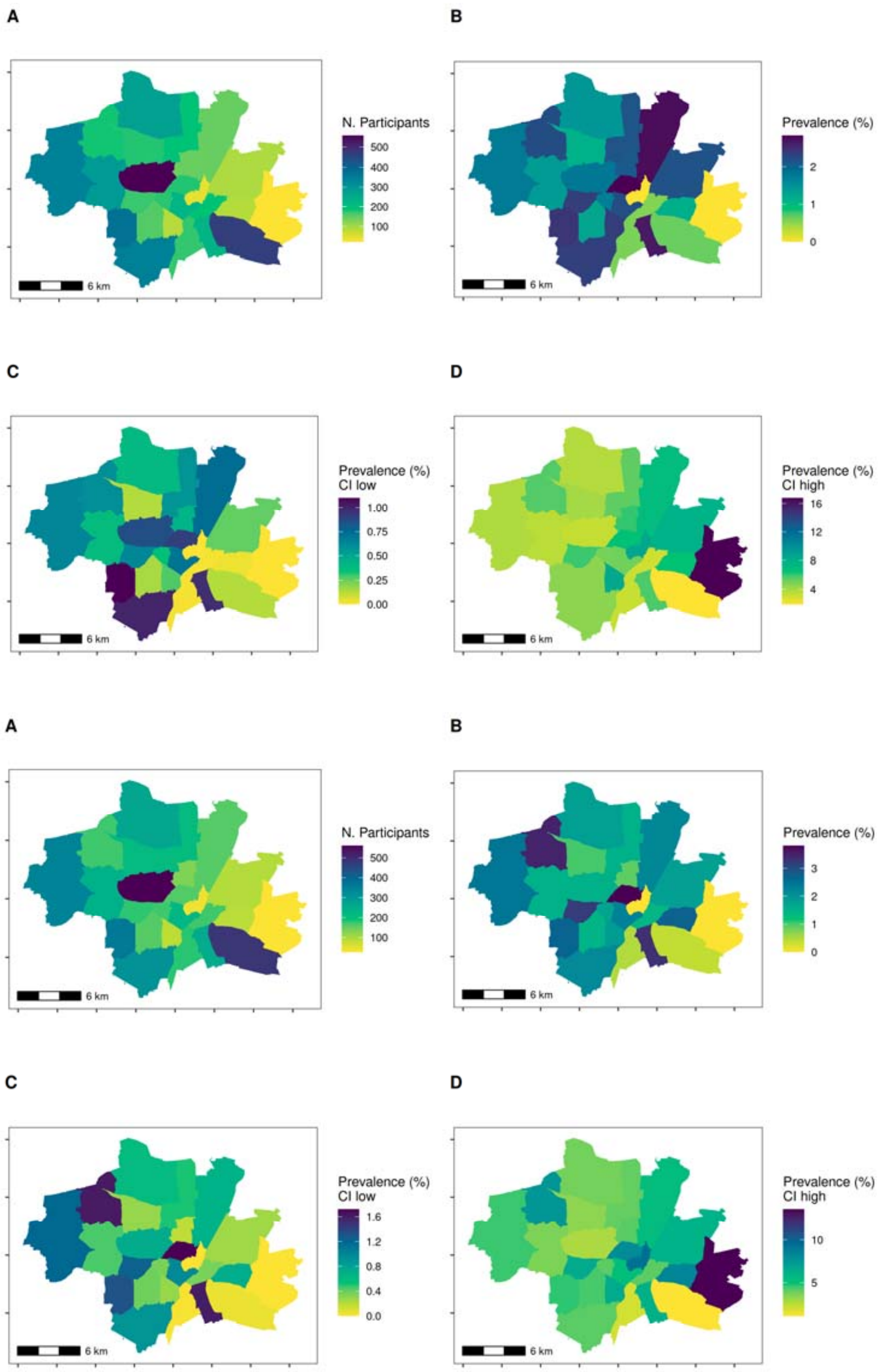

Online Figure S3. Number of participants per city district (**A**), unweighted (upper figure) and weighted (lower figure) prevalence (%) of Ro-N-Ig seropositive samples per city district (**B**) with lower (**C**) and upper (**D**) 95% Confidence Intervals (CI). 95% CI were calculated based on the Poisson Counts for the number of positives among the numbers tested in the district.

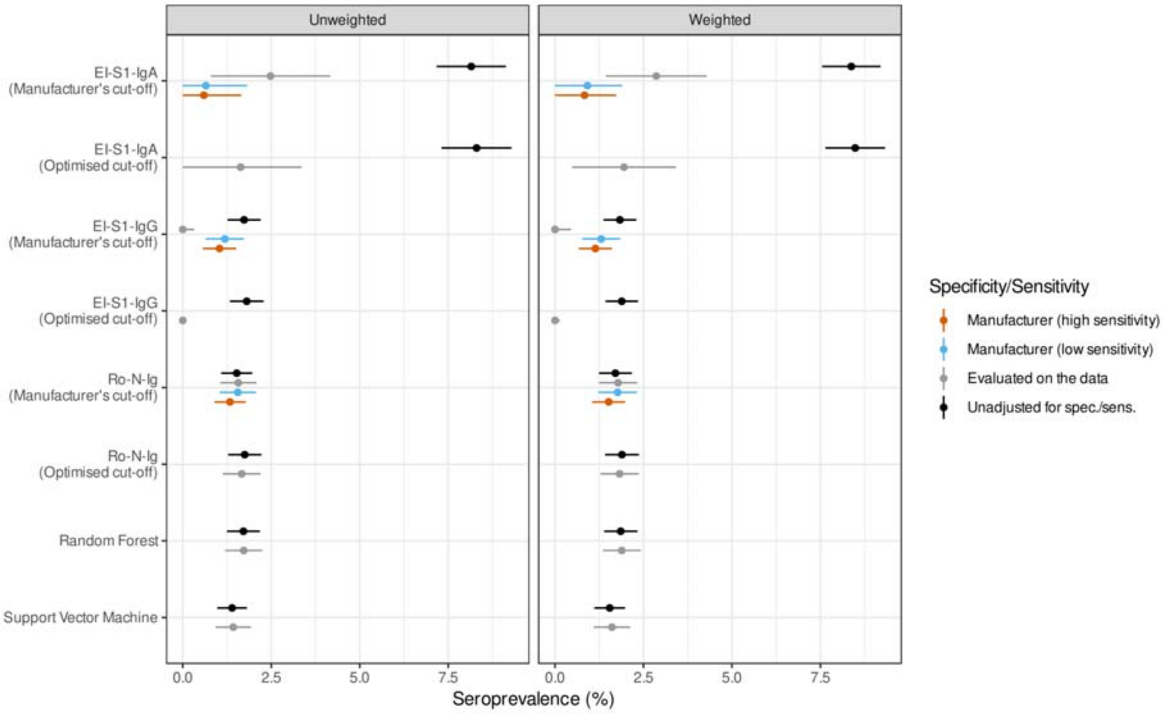

Online Figure S4. SARS-CoV-2 seroprevalence obtained by different classifiers for different specificities and sensitivities. Seroprevalence estimates accounting for the sampling design (right) and not accounting for sampling design (left). Values are adjusted (orange, blue and grey) or unadjusted (black) for the tests' sensitivities and specificities, which are specified in Table S4. In the main manuscript, we report weighted and adjusted Ro-N-Ig seroprevalence with optimised cut-off.

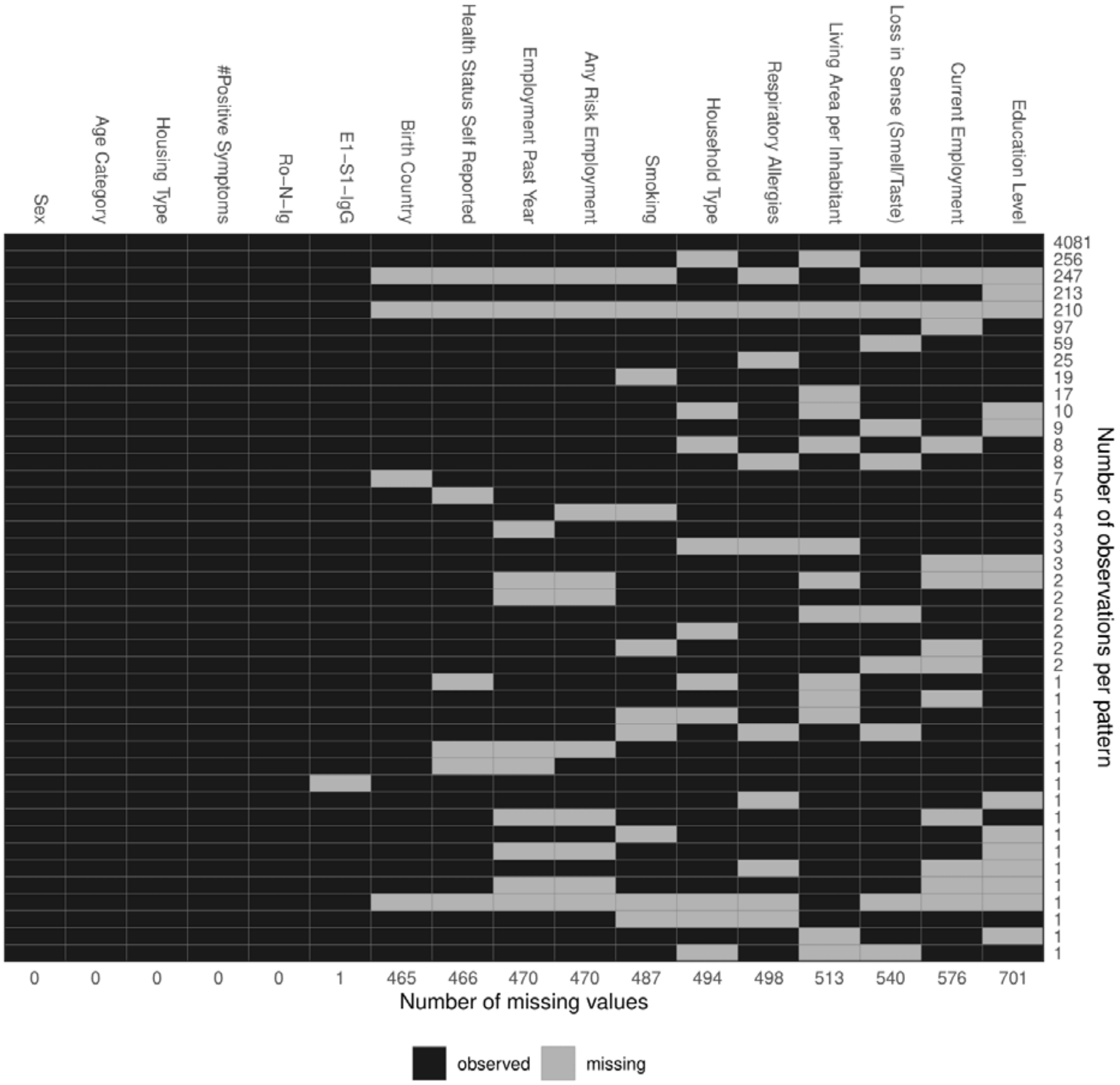

Online Figure S5. Missing patterns observed in our complete analysis data. We observe 4081 out of 5313 individuals have complete information. Our primary serological test (Ro-N-Ig) along with age, sex and the type of housing has complete information for 5313 observations and one individual has missing information on E1-S1-IgG/IgA. Number of positive symptoms is a derived variable based on selection among a list of symptoms mentioned in the questionnaire. Majority of the non-response can be attributed to non-answering of the questionnaire for household and individual characteristics. Missing education level of the participants was the biggest contributor to the missing data.

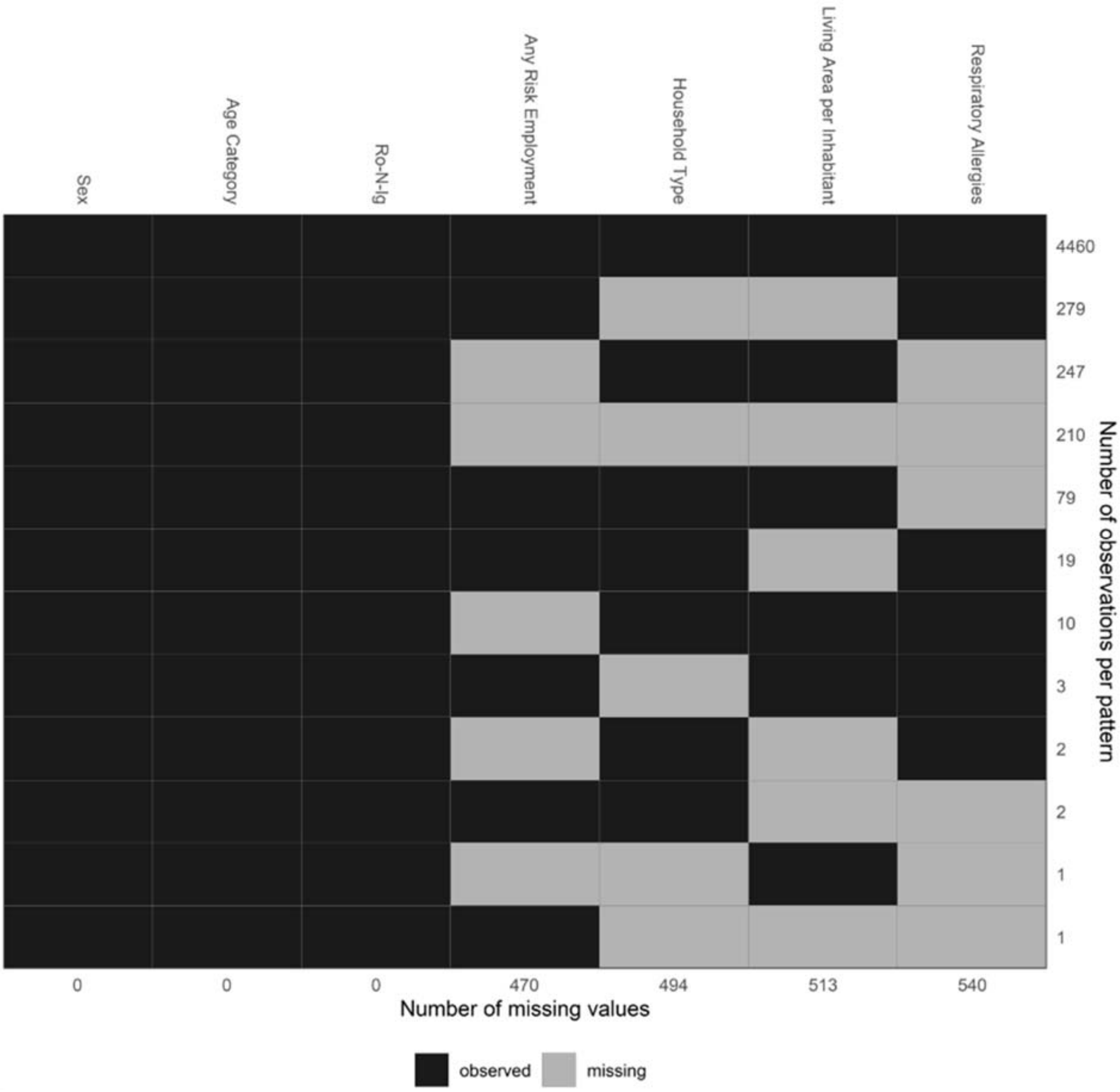

Online Figure S6. Missing patterns observed in our multiple regression analysis data with important covariates only. For this subset of data, we observe 4460 out of 5313 observations have complete information, which is used for obtaining the regression estimates without any imputation (Table S3).

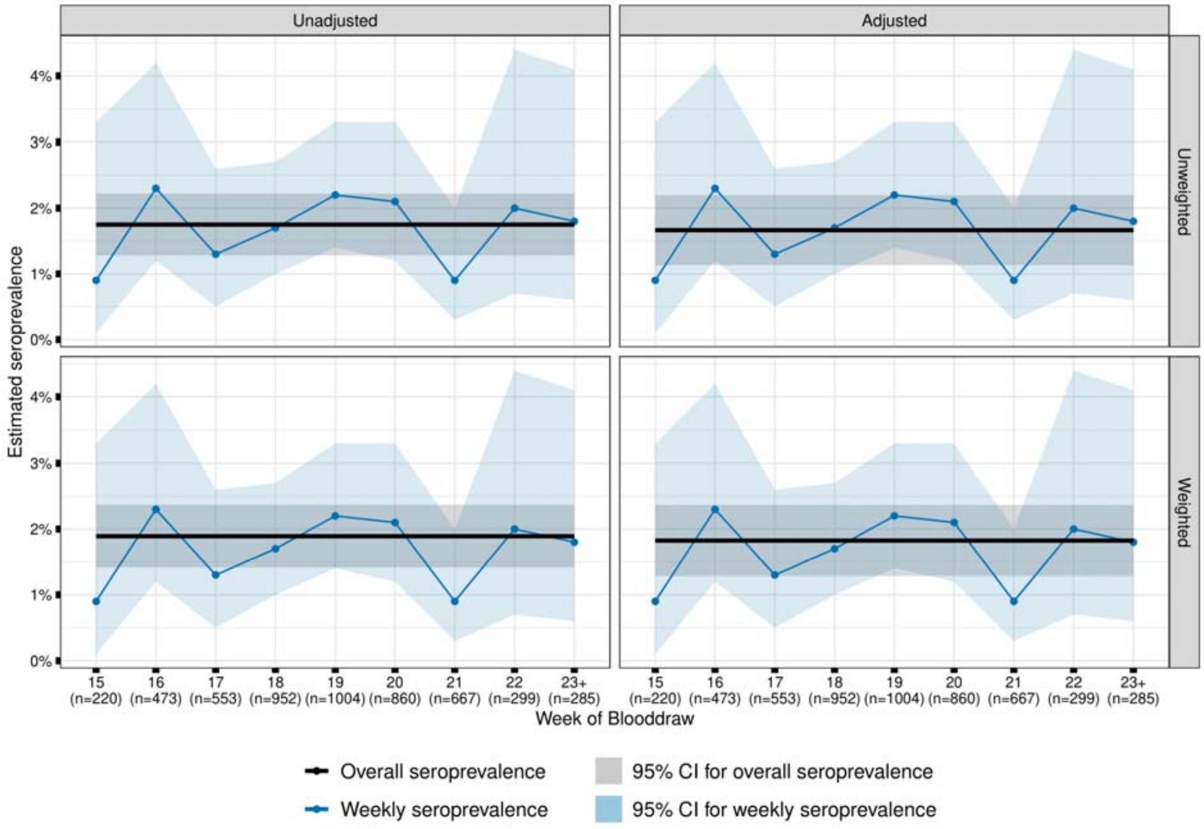

Online Figure S7. Roche N pan-Ig seroprevalence in the KoCo19 study population unweighted (top) and weighted (bottom). The values are either unadjusted (left) or adjusted (right) for sensitivity and specificity of the test method. The weekly seroprevalence estimates are in all scenarios crude estimates without weighting and adjustment for sensitivity and specificity. The 95% confidence interval for the weekly seroprevalence was computed assuming a Poisson distribution to estimate the rate of positive cases.

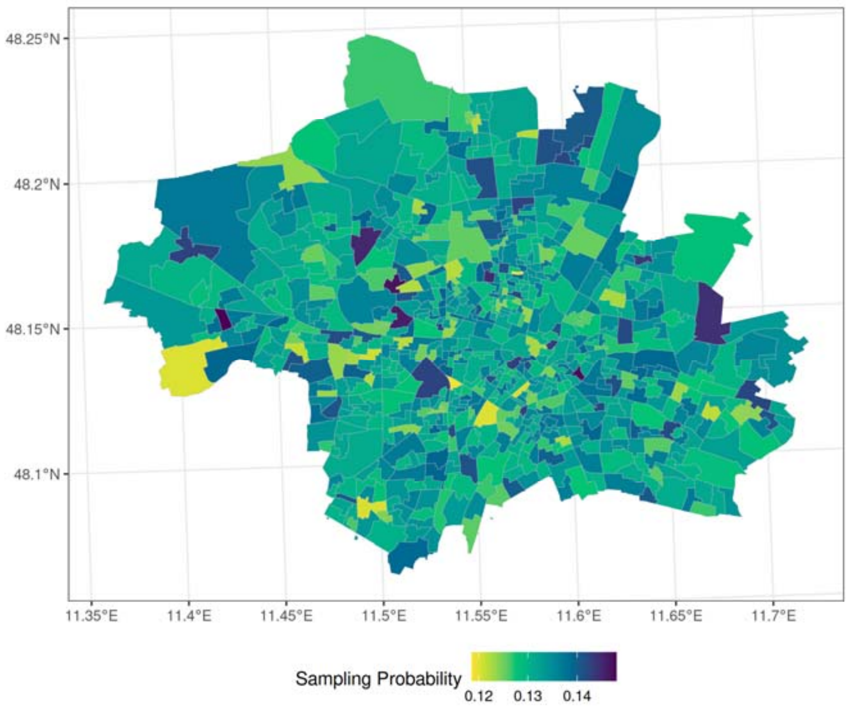

Online Figure S8. Sampling probabilities for Munich constituencies. Given the rejection sampling approach for the selection of the constituencies (see Text S1), we estimated the real probability for each constituency in Munich to be included in the set of 100 starting constituencies for the random walks using a Monte Carlo simulation. Within the samples of 100 constituencies that fulfilled the representativeness requirements, we repeated 5000 times the selection of one sample and computed the sampling probabilities as the percentages of how often a constituency was included in the 5000 Monte Carlo samples. The figure shows that all probabilities are estimated to be in a rather narrow range (between 0.12 and 0.15) and thus approximately equal. The rejection sampling procedure does not induce considerable bias.

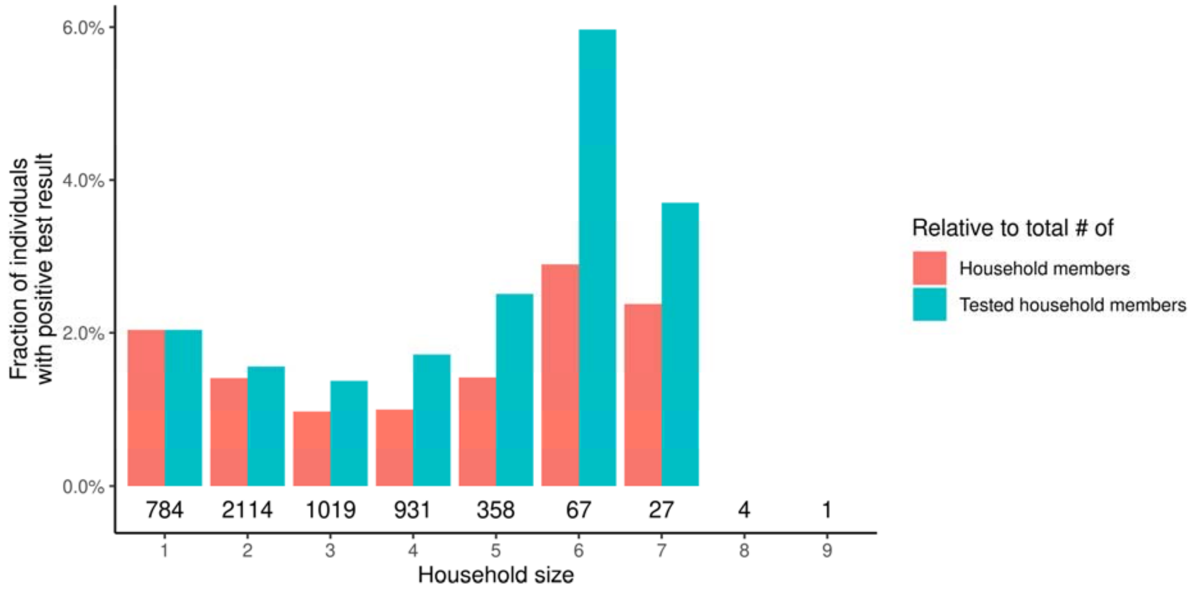

Online Figure S9. Fraction of individuals with positive Ro-N-Ig result for different household sizes. The total number of tested household members (cyan) and the total number of household members (red) was taken as basis.

Online Table S1. Individual characteristics of the KoCo19-study participants comparing telephone interviewees to those answering the online questionnaire.

Characteristics	KoCo-19 Online Questionnaire			KoCo-19 Telephone Interview			Test for Significance [a]
				Individual characteristics			
N		**4902**			**411**		
	$n_{missing}$	*n*	%	$n_{missing}$	*n*	%	***p*-value**
**Sex**	0			0			0.006 [b]
Female		2525	51.5		241	58.6	
**Age**	0			0			0.002 [c]
0–19 years		261	5.3		6	1.5	
20–34 years		1322	27.0		24	5.8	
35–49 years		1505	30.7		37	9.0	
50–64 years		1233	25.2		73	17.8	
65–79 years		504	10.3		172	41.8	
80+ years		77	1.6		99	24.1	
**Country of birth**	465			0			0.035 [b]
Outside Germany		761	17.2		88	21.4	
**Level of education**	685			16			
Still in school		97	2.3		3	0.8	0.002 [c]
<12 years		1103	26.2		283	71.6	
≥12 years		3017	71.5		109	27.6	
**Employment status**	575			1			0.002 [c]
Employed		2825	65.3		86	21.0	
Self-Employed		445	10.3		26	6.3	
Not working[d]		970	22.4		288	70.2	
Others[e]		87	2.0		10	2.4	
**Risk employment** [f]	470			0			0.001 [b]
Yes		3757	84.8		178	43.3	
**General health**	466			0			0.002 [c]
Fair or poor		150	3.4		56	13.6	
Good		1485	33.5		232	56.4	
Very good		2042	46.0		84	20.4	
Excellent		759	17.1		39	9.5	
**Loss of the sense of smell or taste**							
Yes	498			0			0.002 [c]
		40	0.9		13	3.2	
**Respiratory allergies**	537						0.012 [b]
Yes		1283	29.4		96	23.5	
**Smoking status**	575			1			0.002 [c]
Never smoker		2363	53.5		177	43.1	
Ex-smoker		1258	28.5		153	37.2	
Current smoker		794	18.0		81	19.7	
**Face mask use**	469			0			<0.001 [b]
**Almost always**		**3080**	**69.5**		**374**	**91.0**	

[a] Significance test comparing fractions among telephone interviewees to participants who answered the online survey.
[b] Fisher's exact test for count data. [c] Pearson's chi-squared test with simulated p-value (based on 500 replicates); [d] Not working includes unemployed, retired, parental leave, sabbatical, students; [e] Others includes voluntary social year, military service, part-time jobber, reduced working hours; [f] Considered as risk employment for SARS-CoV-2 seropositivity were employees in the: healthcare sector, emergency service, senior homes, airport, public transport, education, sales, social work and other risk jobs. These also include individuals who have been working for the past year at lea.st

Online Table S2. Prevalence of Roche N pan-Ig seropositivity and risk factor analysis adjusted for age and sex using three different methods by individual, household and health characteristics of the KoCo19 study population. The count n represents the number of positives in the specific covariate group and the % symbol denotes the crude prevalence percentage in that group.

Characteristics	Prevalence		Crude (GLM)			Adjusted for Clustering (GLMM)			Bayesian Model Adjusted for Clustering with Multiple Imputation (GLMM)		
**Individual Characteristics**											
	n	%	OR[f]	95% CI[f]		OR[f]	95% CI[f]		OR[f]	95% CI[f]	
**Sex**											
Male	45	1.8	1			1			1		
Female	48	1.7	0.98	0.65	1.48	0.99	0.45	2.20	0.92	0.58	1.50
**Age**											
0–19 years	4	1.5	0.72	0.21	1.84	1.61	0.17	12.50	0.59	0.14	1.90
20–34 years	28	2.1	1			1			1		
35–49 years	29	1.9	0.90	0.53	1.53	2.75	0.64	13.60	0.84	0.43	1.49
50–64 years	19	1.5	0.70	0.38	1.24	0.84	0.17	3.97	0.64	0.33	1.21
65–79 years	12	1.8	0.85	0.41	1.64	3.25	0.51	20.60	0.79	0.36	1.79
80+ years	1	0.6	0.27	0.02	1.27	0.01	0.00	3.20	0.13	0.01	1.15
**Adjusted for Age (continuous) and Sex**											
**Country of Birth**											
Germany	67	1.7	1			1					
Outside Germany	14	1.6	0.99	0.53	1.72	0.54	0.09	2.33	0.87	0.41	1.70
**Level of Education**											
Still in school	2	2.0	0.74	0.11	2.83	0.30	0.01	4.08	0.33	0.04	2.12
<12 years	25	1.8	1			1			1		
≥12 years	53	1.7	0.84	0.52	1.40	0.62	0.17	2.39	0.72	0.36	1.33
**Employment Status**											
Employed	47	1.6	1			1					
Self-Employed	13	2.8	1.89	0.97	3.48	1.20	0.23	5.30	1.80	0.87	3.70
Not working [a]	19	1.5	1.03	0.58	1.75	0.92	0.26	3.02	0.91	0.47	1.70
Others [b]	2	2.1	1.21	0.20	3.99	1.64	0.04	19.50	1.06	0.13	5.75
**Risk Employment [c]**											
No	61	1.5	1			1					
Yes	20	2.4	1.53	0.89	2.50	2.68	0.81	8.71	1.53	0.80	2.79
**Household Characteristics**											
**Housing Type: Building with**											
1–2 aps	27	1.9	1			1			1		
3–4 aps	3	0.8	0.44	0.10	1.24	0.50	0.00	11.60	0.35	0.05	1.38
≥5 aps	63	1.8	0.92	0.59	1.48	1.10	0.23	7.68	0.88	0.45	1.64
Others [d]	0	0.0									
**Household Type**											
Single	12	1.8	1.26	0.61	2.50	1.88	0.16	18.20	1.40	0.33	3.77
Couple	24	1.4	1			1			1		
Family	33	1.7	1.11	0.65	1.94	1.04	0.13	8.92	1.19	0.42	3.19
Others[e]	14	2.9	1.90	0.93	3.75	1.92	0.09	22.40	3.03	0.93	9.63
**Number of Household Members**											
1	16	2.0	1			1			1		
2	33	1.6	0.75	0.42	1.40	0.50	0.07	4.06	0.67	0.21	1.81
3-4	30	1.5	0.68	0.37	1.31	0.46	0.06	3.99	0.59	0.18	1.69
5+	14	3.1	1.35	0.63	2.86	0.83	0.03	10.50	1.96	0.51	8.18
**Living area per Inhabitant (Quartiles)**											
≤ 30 m^2	27	1.6	1			1			1		
30-40 m^2	28	2.3	1.57	0.92	2.71	1.57	0.18	14.30	2.19	0.94	5.11
40-55 m^2	9	0.9	0.65	0.28	1.36	0.67	0.02	8.90	0.69	0.27	1.88
>55 m^2	19	2.1	1.67	0.87	3.16	2.13	0.23	20.70	2.65	0.95	7.27

**Net Family Income (Quartiles)**											
≤ 2500 €	10	1.7	2.00	0.77	5.57	2.13	0.04	312	2.49	0.18	11.5
2500-4000 €	7	0.9	1			1			1		
4000-6000 €	19	1.6	1.87	0.82	4.82	2.11	0.10	272	3.27	0.27	11.1
6000+ €	20	1.8	2.13	0.94	5.45	1.99	0.08	262	3.28	0.33	14.1
**Health Status**											
**General Health**											
Fair or poor	4	1.9	1.58	0.42	4.82	1.45	0.08	18.90	0.97	0.22	3.66
Good	26	1.5	1.14	0.59	2.41	0.97	0.18	5.85	0.79	0.31	1.79
Very good	39	1.8	1.27	0.68	2.56	0.76	0.16	4.19	0.94	0.37	1.86
Excellent	12	1.5	1			1			1		
**Respiratory Allergies**											
No	53	1.6	1			1					
Yes	26	1.9	1.20	0.73	1.90	3.25	1.10	10.30	1.21	0.67	2.16
**Skin Allergies**											
No	67	1.6	1								
Yes	13	2.0	1.20	0.63	2.13	3.17	0.81	12.00	1.28	0.62	2.64
**COPD**											
No	70	1.6	1			1					
Yes	9	2.3	1.48	0.68	2.84	2.81	0.57	11.80	1.58	0.64	3.72
**Autoimmune Disease**											
No	75	1.7	1			1			1		
Yes	6	1.8	1.10	0.42	2.35	1.15	0.14	6.43	0.98	0.30	2.75
**Diabetes**											
No	75	1.6	1						1		
Yes	3	1.4	1.09	0.26	3.06	0.39	0.01	6.04	0.78	0.15	3.17
**CVD**											
No	65	1.7	1			1			1		
Yes	15	1.7	1.24	0.64	2.31	1.01	0.21	4.20	1.16	0.53	2.28
**Obesity**											
No	75	1.7	1			1			1		
Yes	6	2.2	1.40	0.54	3.02	0.51	0.03	4.67	1.12	0.35	3.14
**Cancer**											
No	78	1.7	1			1			1		
Yes	3	1.2	0.81	0.20	2.27	0.56	0.04	4.80	0.64	0.14	2.34
**Loss of the sense of smell or taste**											
No	70	1.5	1			1			1		
Yes	10	18.9	16.3	7.40	32.60	41.3	6.70	231	28.2	9.25	90.22
**Health Behaviour**											
**Smoking status**											
Never	46	1.8	1								
Ex	21	1.5	0.87	0.50	1.47	0.68	0.18	2.32	0.71	0.37	1.29
Current	14	1.6	0.89	0.47	1.59	1.12	0.25	4.41	0.74	0.34	1.49
**Use of facemask**											
<Almost surely	27	1.9	1.20	0.74	1.92	1.68	0.53	5.27	1.25	0.71	2.21
**Almost surely**	**53**	**1.5**	**1**			**1**			**1**		

[a] Not working includes unemployed, retired, parental leave, sabbatical, students; [b] Others includes voluntary social year, military service, part-time jobber, reduced working hours; [c] Considered as risk employment for SARS-CoV-2 seropositivity were employees in the: healthcare sector, emergency service, senior homes, airport, public transport, education, sales, social work and other risk jobs. These also include individuals who have been working for the past year at least; [d] Other types of housing include tents, caravans, or the like; [e] Other household types include shared apartments by e.g., students, subleasing, and assisted accommodation; [f] OR: odds ratio; 95% CI: 95% credible intervals (Bayesian analyses) / 95% confidence intervals (frequentist GLMM).

Online Table S3. Multivariate risk factor analysis for SARS-CoV-2 seropositivity mutually adjusted for all variables in the table.

Characteristics	Adjusted for Clustering (GLMM)			Bayesian Model Adjusted for Clustering with Multiple Imputation (GLMM)		
	N = 4460			N = 5313		
	OR	95% CI		OR[c]	95% CI[c]	
**Sex**						
Male	1			1		
Female	1.00	0.39	2.59	0.93	0.56	1.54
**Age (continuous)**	1.01	0.97	1.04	0.99	0.97	1.01
**Risk Employment** [a]						
No	1					
Yes	1.95	0.52	6.72	1.83	0.90	3.73
**Household Type**						
Single	1.54	0.09	21.10	1.07	0.35	3.27
Couple	1			1		
Family	1.10	0.11	12.20	1.29	0.55	3.08
Others[b]	1.81	0.05	27.10	2.87	0.91	9.27
**Living Area per Inhabitant (Quartiles)**						
≤ 30 m^2	1			1		
30-40 m^2	1.40	0.12	16.2	2.18	0.91	5.32
40-55 m^2	0.68	0.01	11.9	0.74	0.24	2.22
>55 m^2	1.77	0.10	28.0	2.84	0.92	8.98
**Respiratory Allergies**						
No	1			1		
Yes	2.70	0.90	8.55	1.30	0.70	2.45

[a] Considered as risk employment for Covid-19 infections were employees in the: healthcare sector, emergency service, senior homes, airport, public transport, education, sales, social work and other risk jobs. [b] Other household types include shared apartments by e.g., students, subleasing, and assisted accommodation; [c] OR: odds ratio; 95% CI: 95% credible intervals (Bayesian analyses) / 95% confidence intervals (frequentist GLMM).

Online Table S4. Classifier's specificity and sensitivity depending on the manufacturer's or the optimised cut-offs.

Classifier	Cut-off	Specificity Manufacturer	Specificity Evaluated on the Data	Sensitivity Manufacturer (Low High)	Sensitivity Evaluated on the Data
EI-S1-IgA (Manufacturer's cut-off)	1.100	0.924	0.933	0.917/1.000	0.648
EI-S1-IgA (Optimised cut-off)	1.080		0.926		0.648
EI-S1-IgG (Manufacturer's cut-off)	1.100	0.993	0.980	0.875/1.000	0.772
EI-S1-IgG (Optimised cut-off)	1.101		0.978		0.798
Ro-N-Ig (Manufacturer's cut-off)	1.000	0.998	0.998	0.853/0.995	0.855
Ro-N-Ig (Optimised cut-off)	0.422		0.997		0.886
Random Forest			0.998		0.886
Support Vector Machine			0.998		0.845

Online Table S5. Overview on the household size distribution, the number of tested individuals per household and the number of positive tests. Seropositivity is with respect to Ro-N-Ig with optimised threshold.

Number of Inhabitants of the Household	Number of Tested Individuals in Household	Number of Households			
		In Which no Test was Positive	In Which 1 Test was Positive	In Which 2 Tests were Positive	In Total
1	1	768	16	0	784
2	1	223	3	0	226
	2	918	24	3	945
3	1	60	0	0	60
	2	287	8	1	296
	3	120	2	1	123
4	1	44	0	0	44
	2	230	5	2	237
	3	63	2	0	65
	4	51	3	1	55
5	1	12	0	0	12
	2	52	1	1	54
	3	23	0	0	23
	4	19	1	1	21
	5	14	3	0	17
6	1	4	0	0	4
	2	6	0	0	6
	3	6	0	1	7
	4	2	0	0	2
	5	0	1	0	1
	6	2	1	0	3
7	2	2	0	0	2
	4	1	0	0	1
	5	1	0	0	1
	7	1	1	0	2
8	4	1	0	0	1
9	1	1	0	0	1

References

1. Hajek, J. Asymptotic Theory of Rejective Sampling with Varying Probabilities from a Finite Population. *Ann. Math. Stat.* **1964**, *35*, 1491–1523, doi:10.1214/aoms/1177700375.
2. Fuller, W.A. Some design properties of a rejective sampling procedure. *Biom.* **2009**, *96*, 933–944, doi:10.1093/biomet/asp042.
3. Tillé, Y. *Sampling and Estimation from Finite Populations*; Wiley: Hoboken, NJ, USA, 2020.
4. Deville, J.-C., Lavallée, P. Indirect Sampling: The Foundations of the Generalized Weight Share Method. Survey Methodology. Available online: https://www150.statcan.gc.ca/n1/en/catalogue/12-001-X20060029551 (accessed on 28 March 2021).
5. Deville, J.-C., Särndal, C.-E. Calibration Estimators in Survey Sampling. Journal of the American Statistical Association. *J. Am. Stat. Assoc.* **1992**, *87*, 376–382.
6. Deville, J.-C. Variance estimation for complex statistics and estimators: Linearization and residual techniques. *Surv. Methodol.* **1999**; *25*, 193–204.
7. Chauvet, G.; Vallée, A.A. Consistency of estimators and variance estimators in two-stage sampling. *J. Royal Stat. Soc.* **2020**; *82*, 797–815.
8. Sempos, C.T.; Tian, L. Adjusting Coronavirus Prevalence Estimates for Laboratory Test Kit Error. *Am. J. Epidemiol.* **2021**, *190*, 109–115, doi:10.1093/aje/kwaa174.
9. Moreira JP de L, Almeida RMVR de, Rocha NC dos S, Luiz RR. Correção da prevalência autorreferida em estudos epidemiológicos com grandes amostras. Cad Saúde Pública. Available online: http://www.scielo.br/scielo.php?script=sci_arttext&pid=S0102-311X2016001204001&lng=pt&tlng=pt. (accessed on 2 December 2020).
10. Robert Koch Institut. 10. Täglicher Lagebericht des RKI zur Coronavirus-Krankheit-2019 (COVID-19)—Aktualisierter Stand für Deutschland. 2020 Available online: https://www.rki.de/DE/Content/InfAZ/N/Neuartiges_Coronavirus/Situationsberichte/2020-06-13-de.pdf;jsessionid=2A2033699409CB8BA030EB0F22EA0BD4.internet062?__blob=publicationFile. (accessed on 2 December 2020).
11. Olbrich, L.; Castelletti, N.; Schälte, Y.; Garí, M.; Pütz, P.; Bakuli, A.; Pritsch, M.; Kroidl, I.; Saathoff, E.; Noller, J.M.G.; et al. A Serology Strategy for Epidemiological Studies Based on the Comparison of the Performance of Seven Different Test Systems—The Representative COVID-19 Cohort Munich. *medRxiv*. 2021, doi: https://doi.org/10.1101/2021.01.13.21249735.
12. Molenberghs, G.; Verbeke, G. *Models for Discrete Longitudinal Data*. Springer: Verlag, NY, USA, 2005, p. 683.
13. Brooks, M.E.; Kristensen, K.; Van Benthem, K.J.; Magnusson, A.; Berg, C.W.; Nielsen, A.; et al. glmmTMB balances speed and flexibility among packages for zero-inflated generalized linear mixed modeling. 2017. Available online: https://www.zora.uzh.ch/id/eprint/148918. (accessed on 2 December 2020).
14. Erler NS, Rizopoulos D, Lesaffre EMEH. JointAI: Joint Analysis and Imputation of Incomplete Data in R. Available online: https://arxiv.org/abs/1907.10867. (accessed on 2 December 2020).
15. Erler, N.S.; Rizopoulos, D.; Van Rosmalen, J.; Jaddoe, V.W.V.; Franco, O.H.; Lesaffre, E.M.E.H. Dealing with missing covariates in epidemiologic studies: A comparison between multiple imputation and a full Bayesian approach. *Stat. Med.* **2016**, *35*, 2955–2974, doi:10.1002/sim.6944.
16. Verbeke, G.; Molenberghs, G. The use of score tests for inference on variance components. *Biometrics* **2003**, *59*, 254–262, doi:10.1111/1541-0420.00032.
17. Molenberghs, G.; Verbeke, G. Likelihood Ratio, Score, and Wald Tests in a Constrained Parameter Space. *Am. Stat.* **2007**, *61*, 22–27, doi:10.1198/000313007x171322.
